# Degradation of soybean meal proteins by wheat malt endopeptidase and the antioxidant capacity of the enzymolytic products

**DOI:** 10.3389/fnut.2023.1138664

**Published:** 2023-03-03

**Authors:** Jingxiao Fan, Aiying Gao, Chao Zhan, Yuhong Jin

**Affiliations:** ^1^College of Food Science and Engineering, Shandong Agricultural University, Tai'an, China; ^2^Food Inspection Department, Institute for Food and Drug Control (Taian Fiber Inspection Institute), Tai'an, China

**Keywords:** endopeptidase, antioxidant capacity, soybean meal, product proteins, wheat malt

## Abstract

This study investigated the hydrolysis effect of the endopeptidase from wheat malt on the soybean meal proteins. The results indicated that the endopeptidase broke the peptide bonds of soybean meal proteins and converted the alcohol- and alkali-soluble proteins into water-soluble and salt-soluble proteins. In addition, wheat malt endopeptidase did not break the disulfide bonds between proteins but affected the conformation of disulfide bonds between substrate protein molecules, which were changed from the gauche-gauche-trans (g-g-t) vibrational mode to the trans-gauche-trans (t-g-t) vibrational mode. Wheat malt endopeptidase exhibited the highest enzymatic activity at 2 h of enzymatic digestion, demonstrating the fastest hydrolytic rate of soybean meal proteins. Compared with the samples before enzymatic hydrolysis, the total alcohol- and alkali-soluble proteins were decreased by 11.89% but the water- and salt-soluble proteins were increased by 11.99%, indicating the hydrolytic effect of endopeptidase. The corresponding water-soluble proteins had molecular weights of 66.4–97.2, 29–44.3, and 20.1 kDa, while the salt-soluble proteins had molecular weights of 44.3–66.4, 29–44.3, and 20.1 kDa, respectively. The degree of enzymatic hydrolysis of soybean meal reached the maximum at 8 h. The newly created proteins exhibited significantly antioxidant properties, which were inversely related to the molecular weight. Proteins with molecular weight <3 kDa had the highest antioxidant performance with an antioxidant capacity of 1.72 ± 0.03 mM, hydroxyl radical scavenging rate of 98.04%, and ABTS [2,2'-azino-bis(3-ethylbenzothiazoline-6-sulfonic acid)] radical scavenging capacity of 0.44 ± 0.04 mM.

## 1. Introduction

Soybean meal is a by-product from the extraction of soybean oil. Global soybean meal production has showed an upward trend from 2017/18 to 2021/22. According to the U.S. Department of Agriculture's data in November 2022 (1), the global soybean meal production would be 52,839,000 short tons in 2022/23. Soybean meal is cheap and abundantly available, and it has 43% higher protein content (2) and 52% higher antioxidant capacity than soybeans (3), making it a very valuable source of plant protein. However, its application in the food industry is limited by its low palatability, high protein molecular weight, and its potential antinutritional factors and allergens (4). A growing number of studies have found that degrading soybean meal can modify this drawback using physical, enzymatic and microbial fermentation methods. Kong et al. (5) found that the soybean meal treated with steam explosion by 0.7 MPa for 8 min contained less phytic acid and β-polyglycine, resulting in a higher potential utilization value. A study by Wang et al. (6) showed that soybean meal treated with alkaline protease and trypsin also had lower content of antigen b-accompanied soy globulin. Ma et al. (7) found that *Lactobacillus plantarum* and *Enterococcus faecalis* could be used to degrade soybean meal and reached a peak at 30 d, resulting in significantly increased crude protein and fat content. In the study of Yang et al. (8), the mixture of *Aspergillus, Bacillus*, and *Lactobacillus* was used to hydrolyze soybean meal with the aid of alkaline protease fermentation, which effectively reduced its antigenicity of allergens but increased the content of small molecule peptides and total amino acids. Similar to the enzymatic digestion, microbial fermentation uses proteases produced by microorganisms such as fungi and bacteria to degrade soybean meal proteins. It can reduce the anti-nutritional factors and allergens therein in the process, increase the protein and amino acid content (9) and improve the flavor of soybean meal (10).

Plants are one of the main natural sources of antioxidants because they are rich in reactive oxygen detoxification systems that provide different antioxidant compounds such as vitamin C, vitamin E and carotenoids (11). And plants are also rich in proteins that may act as antioxidants through certain amino acids that act as metal chelators and hydrogen donors (12). Protein hydrolysates or bioactive peptides derived from dietary proteins have health-promoting functions, such as the ability to antioxidant, lower blood pressure, and fight cancer (13–15). Researchers have characterized its biological activity and found that the magnitude of activity correlates with its amino acid sequence, composition and molecular weight (16). The product of enzymatic digestion also has excellent antioxidant properties. Kong et al. (17) found that replacing an equivalent amount of soybean meal with enzymatically digested soybean meal in Rex rabbit diets increased the antioxidant capacity in the liver of rabbits. It was also found that enzymatic digestion of soybean meal improved the growth performance, immune function, antioxidant capacity of piglets (18, 19).

There are many methods to determine the antioxidant power of peptides, but the following three were used in this experiment: Ferric Reducing Antioxidant Power, ABTS^·+^ Radical Cation Scavenging Activity, Hydroxyl Radical-Scavenging Activity. FRAP method (ferric reducing antioxidant power), meaning Fe^3+^-TPTZ is reduced to blue Fe^2+^-TPTZ under acidic conditions by antioxidant substances in the sample. FRAP method is simple and fast, and its essence is to substitute equal amount of Fe^2+^. The ABTS method can determine the antioxidant capacity of hydrophilic antioxidants and hydrophobic antioxidants simultaneously and does not depend on the pH value of the medium (20). Hydroxyl radicals act on proteins, nucleic acids, lipids and other biomolecules in the body, causing damage to cell structure and function. Hydroxyl radical scavenging ability is one of the important indicators of antioxidant capacity of samples. These three antioxidant assays can be important methods for characterizing the mechanism of hydrolysate or isolated peptide activity.

Microbial fermentation of soybean meal has gained increased research interest in recent years. However, the utilization of plant-derived proteases for hydrolyzing soybean meal has rarely been reported. Wheat malt is a natural source of complex enzymes, and wheat can synthesize a large amount of endopeptidase during germination by hormone action, which makes the storage protein in wheat extensively degraded (21).

Raman spectroscopy is a molecular analysis technique based on the light scattering effect that yields direct information about secondary and tertiary structural changes of proteins and their aromatic amino acids (22). Therefore, Raman spectroscopy was used in this study to analyze the structure of the product proteins.

In this study, soybean meal was degraded with the wheat malt endopeptidase, and the changes in the content and molecular weights of protein fractions before and after the degradation of soybean meal were investigated. The antioxidant properties of the active hydrolysates in the degradation product were also studied to broaden the application in the food industry.

## 2. Method

### 2.1. Enzymatic digestion of soybean meal and analysis of product composition

#### 2.1.1. Endopeptidase activity assay

The determination method of endopeptidase was modified based on previous studies (23). The 1 mL crude enzyme sample was mixed with 1 mL of acetic acid-sodium acetate buffer (pH 4.5), intensively mixed, and incubated at 50°C for exactly 30 min. Then, the 1 mL of bovine serum albumin solution was added to the reaction mixture and was reacted at 50°C for 4 h. The reaction was stopped by using 1 mL 15% trichloroacetic acid solution and placed at 4°C for 30 min. The mixture was centrifuged at 5,000 r/min for 10 min. The supernatant was mixed with 4 mL of Alkaline copper sulfate solution and left to stand for 30 min at room temperature. The absorbance was measured at 540 nm. The control tube is a 15% trichloroacetic acid solution added immediately before holding to a tube containing a mixture of bovine serum albumin solution, buffer solution, and crude enzyme solution. One unit of the endopeptidase activity was defined as at pH 4.5 and 50°C produce 1 mg of diurea in 1 min per g of dry wheat malt. The enzyme activity can be calculated by the equation as follows:

$$\begin{array}{l} U=\frac{\left({\bar{X}}_{1}-{\bar{X}}_{0}\right)\times 8\times 1000\times \frac{V}{v}}{m\left(1-w\right)\times h} \end{array}$$

Where, U is endopeptidase activity of the sample under test (u); ${\bar{X}}_{1}$ is the concentration of diurea corresponding to the absorbance value of the experimental group (mg/mL); ${\bar{X}}_{0}$ is the concentration of diurea corresponding to the absorbance value of the control group (mg/mL); 8 is the volume of the reaction system is 8 mL (mL); h is the reaction time of enzyme and substrate (min); m is the sampling volume of the sample to be measured (g); V is the volume of fixed volume of crude enzyme solution (mL); v is volume of crude enzyme in the reaction/control group (mL); w is sample moisture content (%).

In the above equation, the diurea equivalents X_0_ and X_1_ were calculated by the following standard curve. The specific procedure of preparing the standard curve was as follows: pipetted 0, 10, 20, 30, 40, 45 mL diurea solution to the test tubes, respectively, and then adjusted the volume to 50 mL by adding distilled water. The solution was mixed with Alkaline copper sulfate solution and allowed to stand for 30 min at room temperature. The absorbance was measured at 540 nm. The linear regression equation of the standard curve was Y = 0.0618x. (*R*^2^ = 0.9993), where X was the concentration of diurea solution (mg/mL) and Y was the absorbance at 540 nm.

#### 2.1.2. Extraction of wheat malt endopeptidase

Wheat malt (purchased from Supertime Malting Co., Ltd, China) was ground and 10 g of wheat malt powder was dissolved in 40 mL of citric acid-disodium hydrogen phosphate buffer (0.1 M, pH 5.2), stirred for 30 min at 4°C, and then fixed to 50 mL. The crude enzyme solution of wheat malt endopeptidase was obtained by filtration through a 0.45 μm filter (Millipore Co., Milford, MA, USA).

#### 2.1.3. Soybean meal digestion by wheat malt endopeptidase

Soybean meal was ground and sieved through a 60-mesh screen. Ten gram of sieved soybean meal and deionized water were mixed thoroughly (mixing volume ratio was 1:4). Then 20 U of wheat malt endopeptidase solution (0.1 M, pH 5.2 citric acid-disodium hydrogen phosphate buffer environment) was added, and finally the volume was fixed to 100 mL. The enzymatic digestion was carried out at 50 °C with the water bath shaking for 0, 2, 4, 6, 8, and 10 h, respectively. After the reaction was completed, the supernatant was collected after centrifugation at 5,000 × g for 20 min. The precipitate was redissolved in 100 mL of deionized water and centrifuged under the same conditions as above. This operation was repeated twice, and the supernatant was collected three times and recorded as the product of water-soluble protein. The precipitate was washed with 0.5 M NaCl solution, 75% alcohol and 0.2% KOH solution for 3 times. After each centrifugation, the supernatant was collected and recorded as the products of salt-soluble protein, alcohol-soluble protein, and alkaline-soluble protein, respectively.

#### 2.1.4. Determination of protein content

GB 5009.5–2010 (24) Kjeldahl method (*F* = 5.71) was used to determine the content of total protein of soybean meal with different degradation time, water-soluble protein, salt-soluble protein, alcohol-soluble protein and alkali-soluble protein.

#### 2.1.5. Sodium dodecyl sulfate polyacrylamide (SDS-PAGE) gel electrophoresis

SDS-PAGE was carried out according to the method of Guo et al. (25).

#### 2.1.6. Raman spectroscopy

The relevant parameters of Raman spectrometer were set as follows: excitation wavelength 785 nm, laser power 80 mW, scan range 400–2,000 cm^−1^, each scan time 70 s, exposure time 70 s, integration 20 times, accumulation of all scans, and at least 6 parallel scans, signal accumulation averaging and plotting output, peak error control within 3 cm^−1^, plotting Raman spectra of soybean meal protein. Peakfit 4.12 software was used to complete the peak-differentiation-imitating analysis. Origin 8.5 software was used for graph production.

### 2.2. Grading of enzymatic products based on molecular weight

The solution was adjusted to pH 4.5 with 2 M HCl solution, then left to stand 1 h, and the supernatant was harvested after centrifugation at 5,000 × g. The molecular weights of soybean meal-hydrolyzed peptides were classified using the Millipore ultrafiltration centrifuge tubes with the interception molecular weights of 30, 10, and 3 kDa.

### 2.3. Determination of the antioxidant properties of enzymatic digestion products

#### 2.3.1. Ferric reducing antioxidant power

The ferric reducing antioxidant power assay was determined using the method reported previously (26).

#### 2.3.2. ABTS^·+^ radical cation scavenging activity

ABTS·+ radical cation scavenging activity was based on the determination method of Pico et al. (27), which adopted the ultraviolet spectrophotometry.

#### 2.3.3. Hydroxyl radical-scavenging activity

The hydroxyl radical-scavenging activity was determined according to the method reported by Zhang et al. (28).

### 2.4. Data analysis

All tests were performed three times, and one-way analysis of variance (one-way-ANOVA) was performed with SPSS 19.0. Duncan's method was used to characterize the significance of differences between factors. Data were expressed as mean ± standard deviation (Mean ± SD). Different lowercase letters indicate significant differences between data at the 95% confidence level.

## 3. Result

### 3.1. Analysis of the degradation mechanism of soybean meal proteins by wheat malt endopeptidase

**Figure 1:**
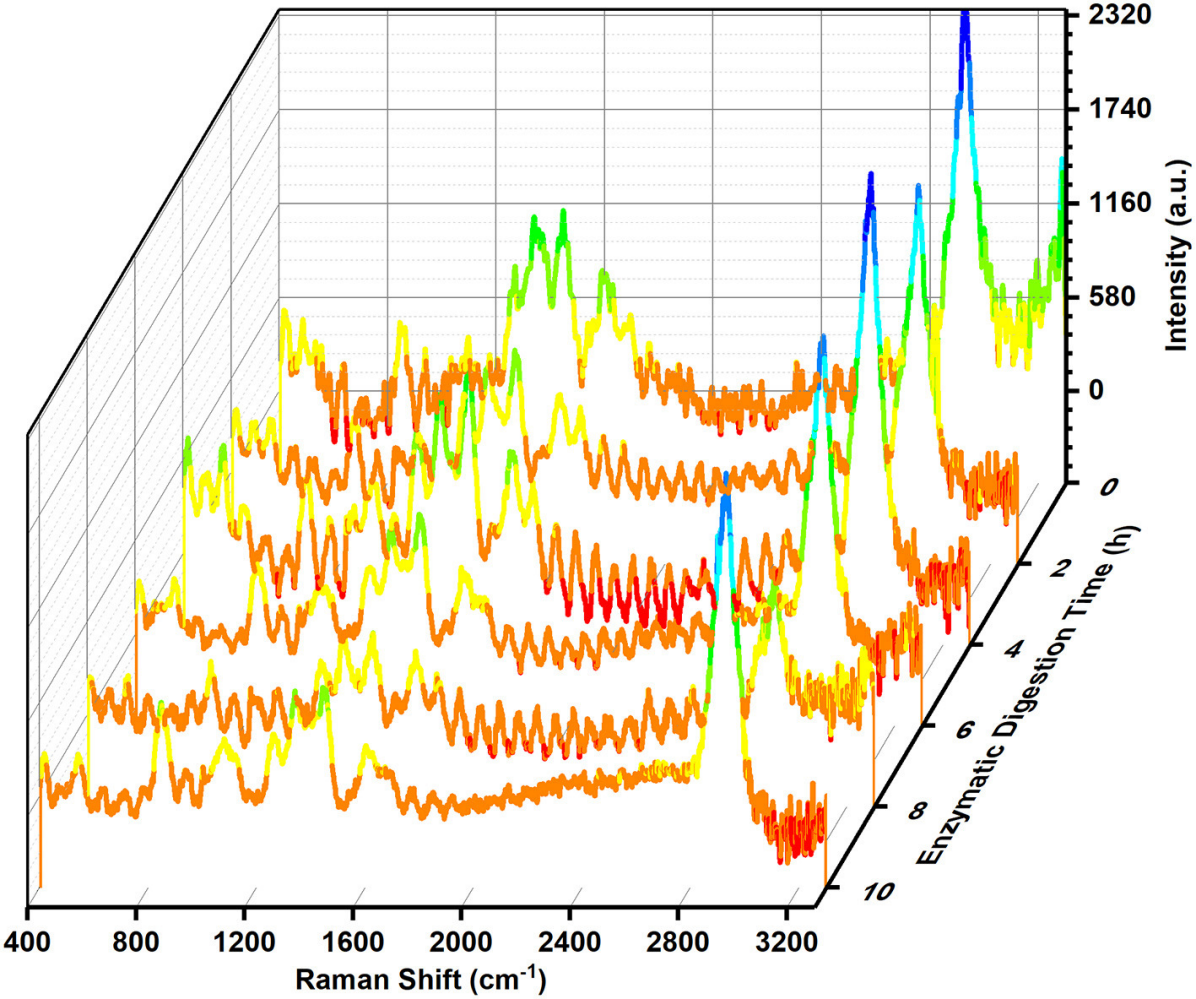
Raman spectra of soybean meal proteins before and after enzymatic digestion by wheat malt endopeptidase.

The Raman spectra of soybean meal before and after degradation by wheat malt peptidase at different time are shown in Figure 1. It could be seen that the soybean meal proteins with different enzymatic digestion time had large spectral peak intensities in the six wave number regions of 600–650, 760–800, 800–900, 1,000–1,050, 1,300–1,400, and 1,500–1,600 cm^−1^. The intensity of the spectral peaks at 600–650 and 1,500–1,600 cm^−1^ varied more at 2 h of enzymatic digestion, those at 760–800 and 1,000–1,050 cm^−1^ varied more at 6 h of enzymatic digestion, and those at 1,300–1,400 cm^−1^ fluctuated more at 2 and 4 h enzymatic digestion. In comparison with previous studies, the wave number regions corresponding to the above peak spectra contained labeled bands for tryptophan (at around 764, 880, 1,347, and 1,556 cm^−1^), phenylalanine (at 1,009 and 1,043 cm^−1^), and tyrosine (at 646 and around 860 cm^−1^), respectively (29). It showed that enzymatic digestion might have caused changes in the number of tryptophan, phenylalanine, and tyrosine residues in soybean meal proteins. Most of the Raman characteristic bands are associated with amide bonds (CO-NH), among which, Amide I (1,600–1,690 cm^−1^) and Amide III (1,230–1,300 cm^−1^) are usually used to predict the protein secondary structures. Amide I region is mainly from C = O stretching vibrations (30, 31). Amide III is mainly associated with C-N stretching and N-H bending vibrations (32, 33). In Figure 1, the soybean meal with different enzymatic digestion time all had strong spectral peaks in the Amide I and III regions. The intensity of the peak spectrum of Amide I region changed significantly after 6 h of enzymatic digestion. While the intensity of the peak spectrum of Amide III region changed significantly within 4 h of enzymatic digestion, there was no significant change after 6 h of enzymatic digestion. This change might be caused by the action of the endopeptidase of malt on the peptide bonds in the polypeptide chain of soybean meal during the enzymatic digestion.

**Figure 2:**
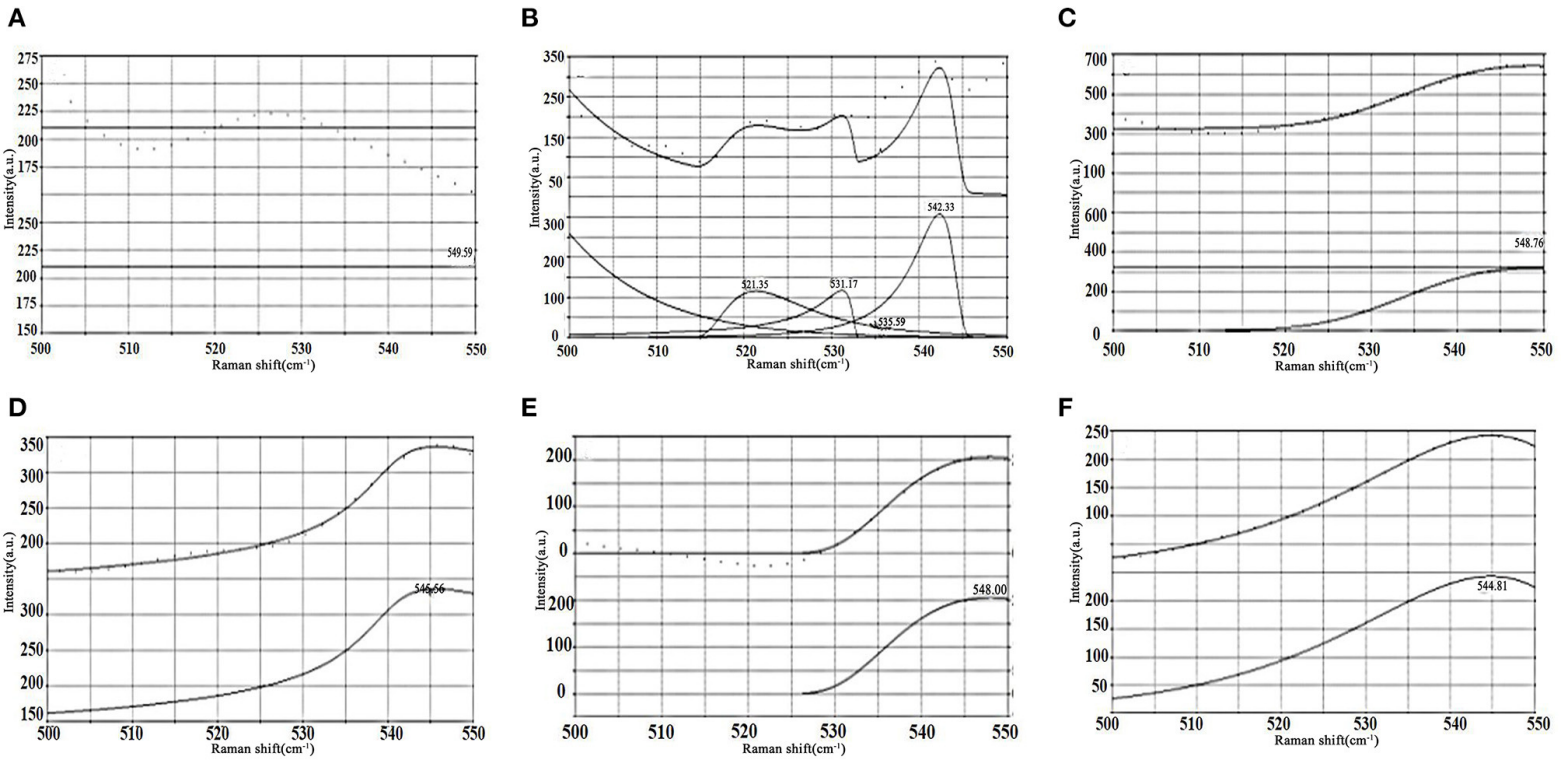
The characteristic bands of disulfide bond in soybean meal protein distinguish the peaks and fitting curves. **(A)** 0 h. **(B)** 2 h. **(C)** 4 h. **(D)** 6 h. **(E)** 8 h. **(F)** 10 h.

Figure 2 shows the fitted Raman spectral band of soybean meal protein in the region of 500-550 cm^−1^. The characteristic spectral band of the disulfide bond is in the range of 500–550 cm^−1^, demonstrating the gauche-gauche-gauche (g-g-g) mode at 500–515 cm^−1^, the gauche-gauche-trans (g-g-t) mode at 515–525 cm^−1^ and the trans-gauche-trans (t-g-t) mode at 525–545 cm^−1^. As demonstrated in Figure 2, the resynthesized fitted spectral lines had a high overlap with the original experimental spectral lines, indicating a good fit and high confidence in the results. There was no peak value of soybean meal protein at 0 h of enzymatic digestion (as Figure 2A). Four peaks were detected at 2 h of enzymatic digestion (as Figure 2B), with two vibrational modes of g-g-t and t-g-t. The vibrational mode of the disulfide bond changed to the t-g-t mode at 4 h of enzymatic digestion (as Figure 2C), and the vibrational mode remained unchanged until 10 h (as Figures 2D–F), indicating that the intermolecular forces between the soybean meal proteins were enhanced and a more stable structure was formed.

The main cleavage site of the wheat malt endopeptidase might be located at the peptide bond with hydrophobic aromatic amino acids (such as phenylalanine and tyrosine) on the carboxyl side (34). It would not break the disulfide bond connection between the substrate protein molecules, but only led to the conversion of the intermolecular disulfide bond conformation from g-g-t vibrational mode to t-g-t vibrational mode.

### 3.2. Capacity and efficiency of wheat malt endopeptidase to degrade soybean meal proteins

**Figure 3:**
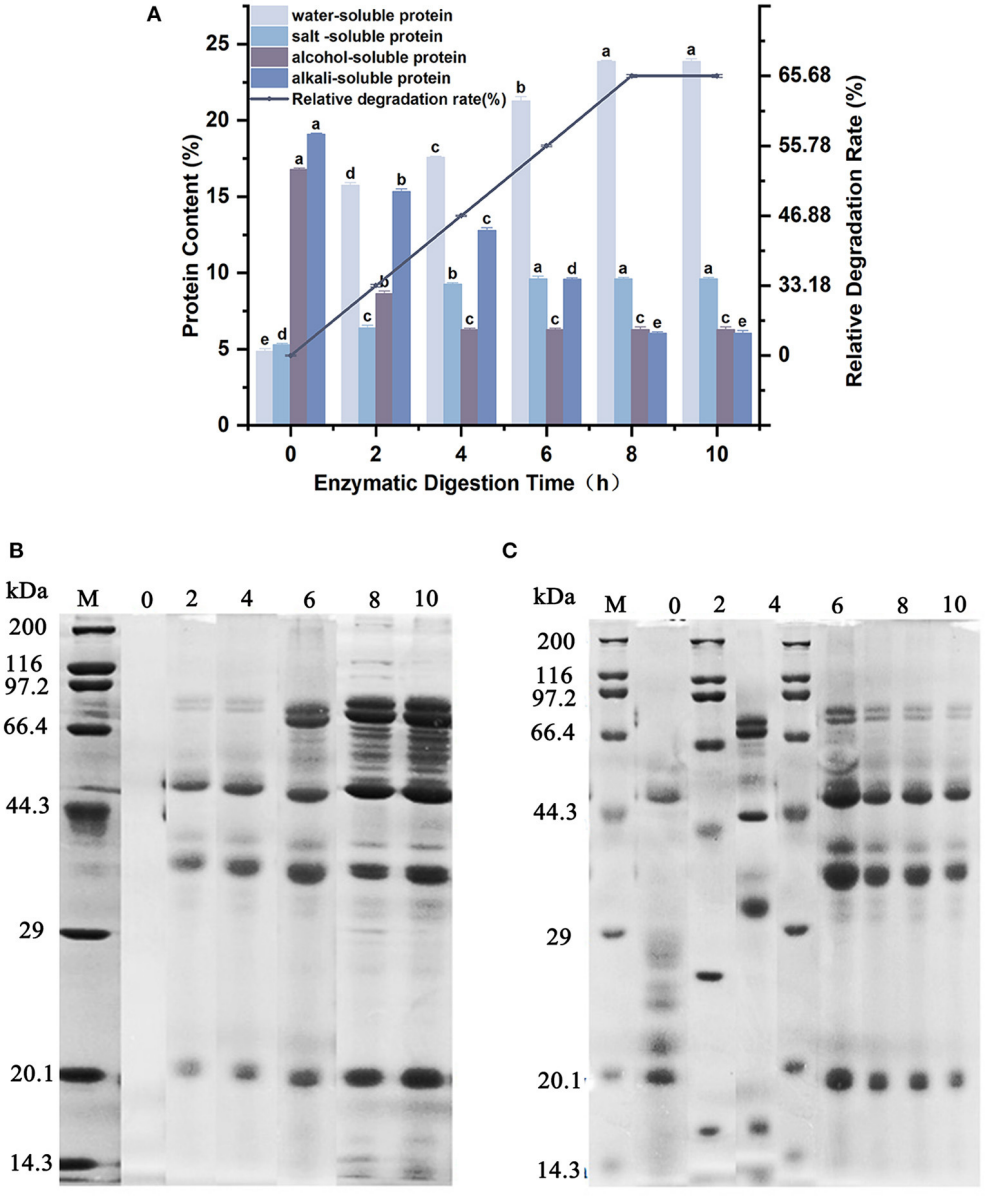
Effect of enzymatic digestion on the enzymatic protein content and composition of soybean meal. **(A)** Changes in protein content of soybean meal during enzymatic digestion. **(B)** Molecular weight distribution of water-soluble proteins in the enzymatic digestion product system. **(C)** Molecular weight distribution of salt-soluble proteins in the enzymatic digestion product system. Different lowercase letters (a–e) indicate significant differences between data at the 95% confidence level.

The content of water-soluble, salt-soluble, alcohol-soluble and alkali-soluble protein fractions in the products of soybean meal degraded by wheat malt endopeptidase was studied, and the molecular weight distribution of water-soluble and salt-soluble proteins with the degradation time is listed in Figure 3. As demonstrated in Figure 3A, the content of alcohol-soluble protein and alkali-soluble protein decreased continuously with time, but the content of water-soluble protein and salt-soluble protein increased continuously with time during the process of 0–10 h when soybean meal was enzymatically digested. After enzymatic digestion for 8 h, 10.50% of alcohol-soluble protein and 13.04% of alkaline-soluble protein were converted to 19.01% of water-soluble protein and 4.32% of salt-soluble protein by the endopeptidase in wheat malt, respectively. The degradation rate was 65.68%. Thereafter, there was no significant difference in the content up to 10 h. The degradation capacity of endopeptidase in wheat malt reached its maximum when the enzymatic digestion proceeded to 8 h. Combined with Figures 3B, C, with the extension of the enzymatic digestion time, the bands of the water-soluble proteins in the enzymatic digestion product system gradually deepened. The number of bands also increased, and the molecular weights of main final product (water-soluble proteins) were mainly located at 20.1, 29.0–44.3, 44.3–66.4, and 66.4–97.2 kDa. The change pattern of the salt-soluble proteins bands was different from that of the water-soluble proteins. While the new salt-soluble proteins were generated, three types of salt-soluble proteins with molecular weights of 66.4–97.2, 29–44.3, and 20.1 kDa were also decomposed during the enzymatic digestion, and the main final product (salt-soluble proteins) showed the molecular weights of 20.1, 29.0–44.3, and 44.3–66.4 kDa.

### 3.3. The antioxidant properties of the enzymatically digested proteins from soybean meal

**Figure 4:**
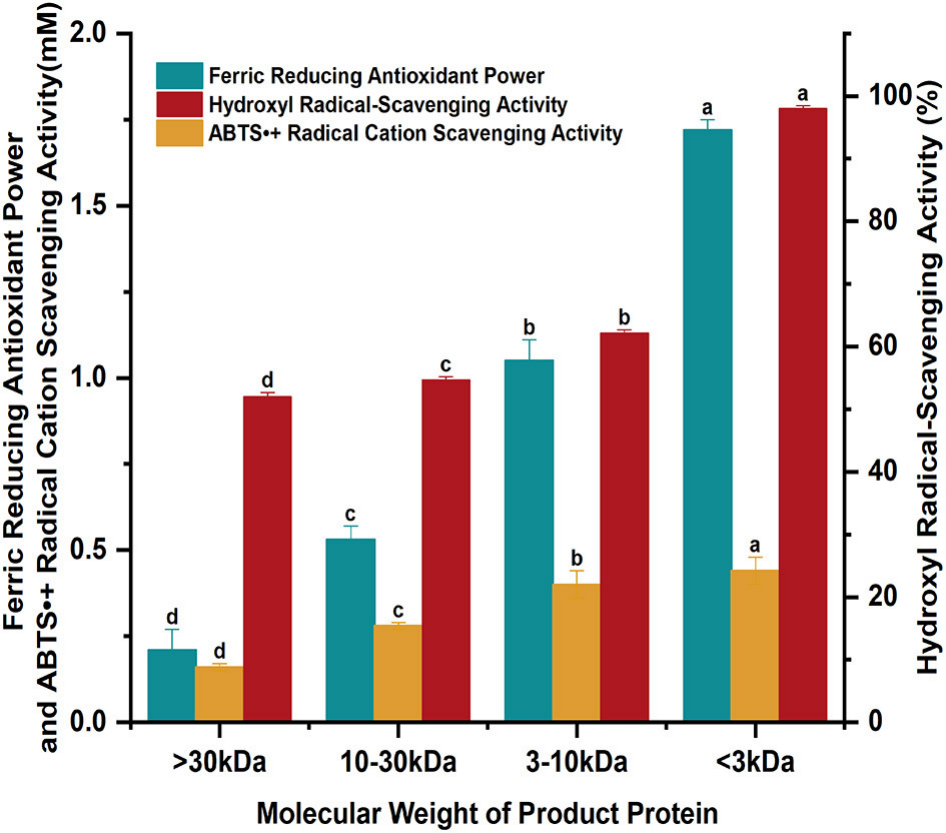
The antioxidant properties of the enzymatically digested proteins from soybean meal.

The ferric reducing antioxidant power, hydroxyl radical and ABTS radical cation scavenging activity of four enzymolytic proteins from soybean meal with different molecular weights were determined and listed in Figure 4. The enzymatic degradation products of soybean meal proteins showed different molecular weights and certain antioxidant capacity. The ferric reducing antioxidant power was 0.21 ± 0.06, 0.53 ± 0.04, 1.05 ± 0.06, and 1.72 ± 0.03 mM for products with molecular weight >30, 10–30, 3–10, <3 kDa, respectively. The hydroxyl radical scavenging activity were 52.03, 54.68, 62.17, and 98.04%; the ABTS radical cation scavenging activity was 0.16 ± 0.01, 0.28 ± 0.01, 0.40 ± 0.04, and 0.44 ± 0.04 mM, respectively. The antioxidant properties gradually increased with the decreased molecular weights. The ferric reducing antioxidant power, hydroxyl radical scavenging activity, and ABTS radical cation scavenging activity of proteins with molecular weight <3 kDa were increased by 719, 88.46, and 175%, respectively, compared with those with molecular weight >30 kDa.

## 4. Discussion

The enzymatic action caused the C = O stretching vibrations, C-N stretching as well as N-H bending vibrations. The number of tryptophan, phenylalanine and tyrosine residues all tended to decrease. Based on the results of Roberts et al. (35), two serine-like proteases were isolated from the dark-induced senescence in wheat leaves. The cleavage site of wheat malt endopeptidase is probably the peptide bond at the carboxyl terminus of the hydrophobic aromatic amino acid. The vibrational modes of disulfide bonds varied significantly at different enzymatic digestion time, and the most drastic changes were observed at 2 h of enzymatic digestion, with two vibrational modes of g-g-t and t-g-t. The vibrational mode of disulfide bonds changed to a single t-g-t mode at the beginning of 4 h of enzymatic digestion, which showed the enhanced intermolecular forces of the enzymolytic protein of soybean meal.

The enzymatic action of wheat malt endopeptidase on soybean meal could convert the alcoholic- and alkaline-soluble proteins in soybean meal to water-soluble and salt-soluble proteins and increase the solubility of proteins. The maximum degree of enzymatic digestion of soybean meal reached the maximum at 8 h, and the fastest enzymatic efficiency was detected at 2 h. The enzymatic digestion products mainly consisted of water-soluble proteins with molecular weights of 66.4–97.2, 29–44.3, and 20.1 kDa, respectively, and salt-soluble proteins with three molecular weights of 44.3–66.4, 29–44.3, and 20.1 kDa, respectively. The enzymolytic products all had certain antioxidant properties, and the highest antioxidant properties were found in the enzymolytic proteins with molecular weight <3 kDa. The ferric reducing antioxidant power was 1.72 ± 0.03 mM, the hydroxyl radical scavenging activity was 98.04%, and the ABTS radical cation scavenging activity reached 0.44 ± 0.04 mM. This was consistent with the study of Shazly et al. (36), where the smaller the molecular weight was, the stronger the antioxidant was.

Fungal and bacterial solid-state fermentation of soybean meal is widely used in soybean meal degradation, where the fungi are mainly *Aspergillus* and *Rhizopus* and the bacteria are mainly *Bacillus subtilis* and *Lactobacillus plantarum* (37). Both fungal and bacterial fermentation can reduce the content of antinutritional factors in soybean meal and increase the content of soluble protein and small peptide (<15 kDa), *in vitro* digestibility, and the antioxidant activity of soybean meal (38, 39). However, bacterial fermentation such as *B. subtilis* and *L. plantarum* was better, it was probably due to the slower rate of fungi such as *Aspergillus* and *Rhizopus*, resulting in a lower microbial activity (40). Compared with other soybean meal degradation methods, the malt endopeptidase enzyme digestion method used in this work was characterized by high safety, easy control of soybean meal degradation process and a short enzymatic digestion time. The enzymatic digestion products have many other advantages over the pre-enzymatic digestion, such as improved soybean meal protein solubility and antioxidant activity. Further study is needed in the future to investigate whether plant-derived endopeptidases such as wheat malt endopeptidase have inhibitory or eliminating effects on potential allergens in soybean meal. In addition, it is necessary to further optimize the enzymatic process of soybean meal in actual production to improve the added value of soybean meal resources.

## Data availability statement

The original contributions presented in the study are included in the article/supplementary material, further inquiries can be directed to the corresponding author.

## Author contributions

YJ conceived and planned the experiments, supervised all the experiments, and analyzed the data. JF and CZ performed all the experiments and analyzed the data. JF wrote the paper. AG contributed reagents, materials, and analysis tools and provided the experimental site. All authors have read and agreed to the published version of the manuscript.
